# Epidemiology and Risk Factors of Functional Constipation in Pregnant Women

**DOI:** 10.1371/journal.pone.0133521

**Published:** 2015-07-24

**Authors:** Wenjun Shi, Xiaohang Xu, Yi Zhang, Sa Guo, Jing Wang, Jianjun Wang

**Affiliations:** 1 The Medical School of Tongji University, Shanghai, China; 2 Huangpu District Maternity and Infant Hospital, Shanghai, China; 3 Department of Gynaecology and Obstetrics, Tongji Hospital, Tongji University, Shanghai, China; The University of Hong Kong, HONG KONG

## Abstract

**Aim:**

To understand the prevalence of functional constipation in pregnant women and to analyze the impact of its risk factors.

**Methods:**

We searched hospital databases for women who were 37–41 weeks pregnant (1698 cases) from July 2012 to January 2014 in four hospitals in Shanghai. We reviewed factors including general data, living and eating habits, psychological history, past history of defecation in the 6 months before pregnancy and defecation after pregnancy. Data were analyzed using SPSS software.

**Results:**

Pregnant women who were more than 35 years old, with a pre-pregnancy body mass index >24, who were highly educated and employed in a sedentary occupation, showed a higher prevalence of functional constipation. Multivariate logistic regression analysis indicated that the prevalence of functional constipation among pregnant women was related to age, pre-pregnancy body mass index, diet, exercise, occupation, psychological factors, threatened abortion in early pregnancy and constipation history.

**Conclusion:**

The prevalence rate of functional constipation in pregnant women was significantly higher than in the general population.

## Introduction

Constipation is one of the most common medical problems in people all over the world. Reduced defecation, lumpy or hard stools and straining are the main manifestations of constipation [1]. The morbidity rate of chronic constipation has increased with changes in diet, lifestyle, psychological and sociocultural factors. Previous studies have found that many factors such as age, gender, socioeconomic status, dietary habits, education level, anxiety, depression and other psychological factors are associated with the prevalence of functional constipation [2–13].The quality of life of patients with constipation is significantly affected in severe cases. Pregnancy is a special period of time for a woman in both physiology and physicality. However, there have been few studies that provide data regarding the prevalence or risk factors for functional constipation in pregnant women. Indirect data suggest that pregnant women may have higher prevalence rates, owing to increased progesterone in body [14], reduced exercise [15] and more protein and fat intake to meet nutritional requirements during pregnancy.

The aim of this study was to understand the epidemiology of constipation and its related risk factors in pregnant women in Shanghai, and to analyze its impact on postpartum outcomes.

## Materials and Methods

### Study selection

From July 2012 to January 2014, 2000 pregnant women were interviewed by trained gynecologists in four hospitals in Shanghai (Tongji Hospital of Tongji University, Ruijin Hospital, Huangpu District Maternity and Infant Hospital, Fengxian District Central Hospital). All respondents provided the written informed consent to participate in this study, and this survey had got the approval of the Tongji Hospital's ethics review committee. And the pregnant women completed questionnaires under the supervision of gynecologists who provided explanations if necessary.

### Diagnostic criteria

Functional constipation was diagnosed using the Rome III criteria and must have included the presence of at least two of the following symptoms for at least one quarter of defecations for the last 12 weeks: straining; lumpy or hard stools; sensation of incomplete evacuation; sensation of anorectal obstruction; manual maneuvers to facilitate defecation; fewer than three defecations per week. Loose stools are rarely present without the use of laxatives [16]. Irritable bowel syndrome and abnormalities of the anal region (fistula and piles) were excluded.

### Inclusion criteria

Data were collected from women who were 37–41 weeks pregnant, aged 18–45 years, willing to comply with the study protocol, mentally healthy and able to communicate and understand normally.

### Exclusion criteria

Exclusion criteria were as follows: having difficulty with mobility, communication or poor compliance; history of cancer, rheumatic diseases, blood diseases, endocrine diseases or digestive tract diseases; medication with salbutamol, ritodrine-hydrochloride, magnesium sulfate and other drugs taken to prevent delivery before 37 weeks of pregnancy; multiple gestations.

### Questionnaires

Data collected included general data (e.g., age, education, occupation, income, height and weight), daily living conditions (e.g., eating, drinking, activity and sleep pattern), psychological factors, past history of defecation in the 6 months before pregnancy and after pregnancy, taking medication or health products to assist defecation and history of threatened abortion during early pregnancy. Each individual completed the questionnaires was interviewed by telephone after pregnancy about more information (e.g., recovery of gastrointestinal function, time of first defecation after delivery, method of delivery, hemorrhage, birth weight of baby) to analyze the impact of constipation on postpartum outcomes.

### Statistical methods

All questionnaire results were recorded and checked by two reviewers via the EpiData 3.1 database, and were then entered into the computer, using SPSS 19.0 statistical software for statistical analysis. We compared prevalence rates of different groups by chi-squared (χ2) text and risk factors of constipation were analyzed by multivariate logistic regression analysis.

## Results

Among the 2000 respondents, a total of 1698 respondents (84.9%) were included in the study according to the criteria. Of them, 221 (13.01%) were classified as having functional constipation using the Rome III criteria. Pregnant women who were more than 35 years old, with a pre-pregnancy body mass index >24, who were highly educated and employed in a sedentary occupation, showed a higher prevalence of functional constipation (Table 1). Multivariate logistic regression analysis indicated that the prevalence of functional constipation among pregnant women was related to age, pre-pregnancy body mass index, diet, exercise, occupation, psychological factors, threatened abortion in early pregnancy and constipation history.

**Table 1:** The impact of associated risk factors (age, education, occupation and income) on pregnant women with constipation.

Associated factors		Prevalence (%)	OR	95%CI of OR	*P*
Age (years)	<25	1.22	1.000		
	25~34	8.22	1.744	1.227–2.478	0.002
	35~45	22.49	3.010	2.224–4.076	0.000
Education	Junior or lower	4.85	1.000		
	Senior	4.24	0.918	0.630–1.338	0.656
	Tertiary	13.44	2.126	1.146–3.942	0.015
	Higher	27.22	5.150	2.213–11.989	0.000
Occupation	Business	28.55	1.000		
	Service industry	5.59	0.801	0.537–1.193	0.641
	Farming and technology	1.68	0.482	0.286–0.811	0.011
				
				
				
BMI	<18.5	4.16	1.000		
	18.5~24	6.65	1.373	0.939–2.008	0.101
	>24	27.34	3.265	1.387–7.683	0.004

### Age and constipation

Data were collected from 1698 pregnant women with a mean age of 28.12 ± 2.80 years and an age range of 18 to 45 years. Prevalence of constipation in women over 35 years of age was 22.49%, in women aged 25 to 34 years and less than 25 years was 8.22% and 1.22%, respectively. The prevalence of defecations in all age groups were significantly different (P < 0.05). In conclusion, the prevalence rate of functional constipation increased with age.

### Education and constipation

In our study, there were 360 pregnant women with Master’s or higher degrees, and this group showed a higher prevalence of constipation (27.22%). However, in the less educated women, the prevalence of constipation was lower (junior or lower: 4.85%, senior: 4.24%, tertiary: 13.44%).The differences were statistically significant (P < 0.05). Therefore, education may be a factor that influences the prevalence of constipation in pregnant women.

### Occupation and constipation

We divided occupation into three classes: business, service industry, farming and technology. Pregnant women who worked in business showed a higher prevalence of constipation (28.55%), while women employed in service industry, farming and technology had significantly lower prevalence rates (5.59% and 1.68%, respectively). The differences were statistically significant (P < 0.05), showing that more sedentary occupations were related to a higher prevalence of constipation.

### Pre-pregnancy BMI and constipation

Prevalence rate of functional constipation increased with pre-pregnancy body mass index (BMI >24, 27.34%; BMI 18.5–24, 6.65%; BMI <18.5, 4.16%). The difference was statistically significant (P < 0.05).

### Multivariate logistic regression analysis for risk factors of functional constipation in pregnant women

Multivariate logistic regression analysis(Table 2)showed that pregnant women who consumed the most fruit and vegetables and who took regular exercise were protected from functional constipation (P < 0.05). Whereas, older age, BMI >24, higher education, employment in sedentary occupations, spicy food intake, stress, depression, threatened abortion during early pregnancy and constipation history were all risk factors of functional constipation (P < 0.05).

**Table 2:** Multivariate logistic regression analysis of the risk factors of constipation in pregnant women.

Factors	B	S.E	P	OR	95%CI of OR
Age(years)	0.701	0.092	0.000	2.013	1.605~2.402
BMI before pregnancy	0.493	0.165	0.005	1.445	1.105~2.126
Education	0.263	0.075	0.002	1.231	0.963~1.423
Occupation	0.639	0.156	0.001	2.065	1.102~2.325
Dietary and living habits					
Vegetables	-0.065	0.038	0.012	0.384	0.021~0.596
Fruit	-0.328	0.101	0.007	0.636	0.254~0.921
Spicy food	0.438	0.079	0.006	1.513	1.230∼1.653
psychological factors					
Insomnia	0.525	0.559	0.789	1.086	0.198∼1.230
Emotional stress	0.398	0.538	0.023	1.568	1.251∼1.647
Depression	0.859	0.429	0.029	1.905	1.206∼1.968
Other factors					
Exercise	-0.579	0.26	0.018	0.612	0.263~0.932
Threatened abortion during early pregnancy	0.655	0.192	0.006	1.816	1.621∼2.752
Constipation before pregnancy	0.685	0.526	0.016	1.512	1.203∼2.437

### The impact of functional constipation in pregnant women on their modes of delivery

We interviewed 1698 pregnant women by telephone about their modes of delivery. The results were divided into two groups according to whether they had functional constipation or not (Tables 3 and 4).

**Table 3:** The impact of functional constipation in pregnant women on their modes of delivery.

Groups	n	Cesarean n (%)	Spontaneous labor n (%)	Forceps delivery n (%)	Fetal macrosomia n (%)	Postpartum hemorrhage n (%)
Constipation	221	148* (66.97)	67* (30.32)	6 (2.71)	16 (7.24)	8 (3.62)
Non-constipation	1477	403 (27.29)	1018 (68.92)	56 (3.79)	83 (5.6)	56 (3.79)

*Compared with the non-constipation group, χ^2^ = 138.24, P < 0.05.

**Table 4:** The impact of functional constipation in pregnant women on postpartum recovery of gastrointestinal function.

	Non-constipation group (N = 1477)	Constipation group (n = 221)	P
Time of first defecation after cesarean (h)	25.24 ± 2.15	36.35 ± 2.45	0.00
Time of first defecation after normal vaginal delivery (h)	13.32 ± 1.56	25.15 ± 1.65	0.00
#Development of postpartum hemorrhoids n (%)	198 (13.40)	52 (23.52)	0.00

^#^Excluding women with hemorrhoids during pregnancy.

Table 3 clearly shows the proportion of Cesarean sections was greater in women with functional constipation (66.97%) compared to women in the non-constipation group (27.29%). Recovery of gastrointestinal function and the time of first defecation after delivery were longer for women with functional constipation, regardless of their mode of delivery. The prevalence of postpartum hemorrhoids was higher in women with constipation compared to non-constipated women (23.52% and 13.40%, respectively). However, there was no significant difference in the incidence of postpartum hemorrhage between the two groups. In terms of birth weight in newborns, the two groups showed no significant difference in the incidence of increased birth weight in newborns. And there was only one case of a birth defect (ear neoplasm) in 1698 newborns.

## Discussion

This is the first study specifically designed to evaluate the prevalent of constipation and its related risk factors in pregnant women in China according to the Rome III criteria. Constipation is influenced by many factors. When constipation does not have an anatomical or physiological cause, then it is considered to be functional constipation. It is a common intestinal disease, especially found in the elderly and in middle-aged women. However, with increased sociocultural changes, higher stress levels and changes in diet, patients are being diagnosed with functional constipation at a younger age. In our study, the overall prevalence of functional constipation as defined by Rome III was 13.01%. Consistent with the previous studies [2–13], we found that constipation was associated with age, diet, exercise, occupation and psychological factors.

Pregnancy is an extremely important period of time for a woman. Extensive physiological, biochemical and dietary changes occur during pregnancy. The body secretes a large amount of progesterone which causes decreased muscle tone and lower motility of the gastrointestinal tract [14]. As the uterus grows during the third trimester of pregnancy, especially after the fetal head becomes engaged in the pelvis, the lower part of the gastrointestinal tract and rectum become compressed, which can cause the formation of hemorrhoids [17]. Some women have difficulty with constipation prior to pregnancy and the condition becomes worse during pregnancy due to reduced physical activity and fear of pain during defecation. Furthermore, nutritional requirements are increased during pregnancy, often with increased protein and fat intake in comparison with vegetable intake, which can lead to infrequent bowel movements and difficulty passing stools. Many heavily pregnant women tend to rest all day, which can decrease gastrointestinal digestion activity and cause bloating of the abdomen and constipation [15]. Our data indicated that pre-pregnancy body mass index, threatened abortion in early pregnancy and constipation history were also risk factors in pregnancy.

### Epidemiology of functional constipation in pregnant women

Our study showed that the prevalence of functional constipation among pregnant women in Shanghai (13.01%) was higher than among the general population (6.00%) [7]. We also found that the prevalence of functional constipation was higher in women over 35 years of age, which is consistent with two other previous studies (one was in the United States [18] and the other was in China [19]). Both of these studies reported that the prevalence of chronic constipation increased with age.

In recent years, increasing levels of stress and the improvement of educational level and social status, have led women to postpone childbearing and primiparas until over the age of 35 years or later. While many women over 35 years of age have normal pregnancies, those over 35 years old have special considerations for pregnancy with the decline of physiology and degeneration of the pelvis and ligaments, the risk of gestational diabetes, high blood pressure, miscarriage and other complications are undoubtedly increased. Furthermore, older women generally suffer more emotional stress than younger women during pregnancy. Xin et al. [20] showed that mood disorders may increase the prevalence of constipation.

At present, the relationship between occupation and functional constipation is controversial. A previous study reported that the prevalence of functional constipation in farmers was the highest [21], whereas another research study showed that teachers, civil servants and white-collar workers were those diagnosed with constipation due to their long-term sedentary occupations [22]. Nevertheless, these studies have all clarified that there is a relationship between chronic constipation and occupation. Our study showed that more sedentary occupations are related to a higher prevalence of constipation, probably due to the long-term lack of physical activity in sedentary occupations.

Previous studies have also shown different findings regarding the relationship between education and functional constipation. One study showed that the prevalence of functional constipation is higher among the population with primary school education in comparison with other populations in Tianjin in China [23]. However, another study identified that education had no relationship with constipation [24]. In our research, we found that highly educated pregnant women were diagnosed more often with defecation disorders, which may be associated with their diet, age, low physical activity, high work pressure and emotional stress at work.

Our study showed that the prevalence of functional constipation in women with a pre-pregnancy BMI >24 was higher than other BMI ranges (27.34%). Overweight women may prefer a diet high in protein and fat with a lower intake of vegetables, which can lead to low dietary fiber intake and dyspepsia. Furthermore, these women may not be able to digest food easily due to low activity levels. Therefore, the incidence of constipation may be increased after pregnancy due to a low fiber diet and less physical activity. All of the previous results are consistent with other studies [25–27].

### Analysis of the risk factors of functional constipation in pregnant women

After pregnancy, almost all women take less exercise, which is one of the most common causes of constipation. In addition, in the third trimester of pregnancy, some pregnant women avoid straining while defecating as they are concerned about causing the fetus to descend, which leads to further constipation. Studies have shown that pregnant women should take proper exercise such as walking, gymnastics and so on. On the contrary, if there is no moderate physical activity then body energy may not be consumed due to low metabolic rate and decreased physical activity [28]. Though some studies have reported physical activity appears to be unrelated to the risk of constipation [29], higher physical activity has been associated with improved quality of life [30]. In this study, multivariate regression analysis showed that moderate exercise was a protective factor for the prevention of functional constipation in pregnancy.

Constipation and dietary habits are closely related, and low dietary fiber intake often causes constipation. The diet of pregnant women is not considered to be a balanced diet if it includes a high proportion of protein and fat. It is advisable that pregnant women consume a high fiber diet containing foods such as fruit and vegetables. In addition, pregnant women may want to avoid spicy food and alcohol to prevent bowel disease and any adverse effects on the development of their fetus [31,32]. In our multivariate regression analysis, intake of fruit, vegetables and other foods containing fiber was found to be a protective factor for preventing constipation during pregnancy.

Researchers have shown that hospitalized pregnant women faced more emotional stress and changes in their environment which are also important causes of constipation [33,34]. Some hospitalized pregnant women feel negative emotions such as irritability, anxiety, tension, depression and anger, which may lead to neurological disorders. In China, almost all deliveries take place in hospital, which means that pregnant women have to share a room with other patients. Therefore, their emotions will be greatly influenced by environmental noise and unfamiliar living conditions. Multivariate regression analysis in our study showed that emotional stress and low mood are risk factors for functional constipation during pregnancy.

Some women take laxatives or colon hydrotherapy in order to lose weight, which causes sensitivity of the rectum to become diminished and unresponsive [35]. And some women have constipation symptoms without any obvious reasons prior to their pregnancy. In our study, multivariate regression analysis showed that a past history of constipation is also a risk factor for constipation in pregnant women.

### The impact of functional constipation in pregnant women on their mode of delivery

Our study showed that the Cesarean section rate in pregnant women with functional constipation (66.97%) was higher than in the other methods of delivery (27.29%).

We also found that postpartum recovery of gastrointestinal function was affected by constipation. Women with constipation suffered for longer with disrupted gastrointestinal function, whether they give birth by Cesarean section or by vaginal delivery. Furthermore, the prevalence of postpartum hemorrhoids in women in the constipation group was higher than in the non-constipation group (23.52% and 13.40%, respectively). There were 85 patients with hemorrhoids before pregnancy (5.01%) and 409 patients had hemorrhoids during pregnancy (24.09%). In the 221 pregnant women with constipation, 150 patients (67.87%) developed hemorrhoids during pregnancy and in 52 patients (23.53%) hemorrhoids occurred after delivery.

Although the data showed the proportion of Cesarean sections in women with functional constipation was higher than in non-constipated women, we cannot confirm that constipation is a risk factor of Cesarean section because telephone interviews were not completed for all study patients, and some patients or their families were not able to provide clear information regarding Cesarean section in the interviews. Furthermore, the factors affecting the Cesarean section rate are variable e.g., age, BMI, fetal size, family factors, genetic factors, obstetric complications, abnormal labor and so on. Consequently, there is not enough evidence to show that constipation is responsible for an increased incidence of Cesarean section. It is a limitation of our study. Further analysis and investigation is required to provide detailed statistics to determine the impact of functional constipation in pregnant women on their mode of delivery.

We reviewed the reasons why pregnant women are more susceptible to developing hemorrhoids. Firstly, the blood flow to the pelvic area increases during pregnancy. Secondly, in the third trimester of pregnancy, the growing uterus exerts pressure onto the pelvis, so that hemorrhoid blood vessels are blocked from returning. Some pregnant women always strain while defecating and this increases abdominal pressure and causes blood stasis in the rectum and anus venous plexus, which can induce or aggravate hemorrhoids. Finally, lower extremity edema and anal varices may occur in many women in the third trimester of pregnancy. Unfortunately, when delivering a baby, abdominal pressure increases so sharply that hemorrhoids become worse such as edema, valgus, prolapse or incarceration. All of these conditions may cause intense suffering to pregnant women and do not assist with postpartum recovery.

Constipation increases the level of toxins in pregnant women, causing metabolic disorders, endocrine disorders and an imbalance in trace elements. At the same time, accumulation of toxins in the intestines may have serious negative effects on fetal development, which may lead to fetal malformation, although such reports are rare. In our study, only one case in 1698 had a minor birth defect.

Another limitation is that estimates of fiber intake were approximate. However, we could not obtain complete and reliable information for a more accurate measure.

In conclusion, functional constipation is a common gastrointestinal disease in pregnant women. Our study showed that the prevalence of functional constipation among pregnant women was 13.01% in Shanghai, China. A high fiber diet and moderate exercise were factors for preventing constipation during pregnancy. Pathogenesis of constipation is caused by multiple factors [36], which make constipation difficult to treat. Further research into the etiology of constipation will help to reduce the incidence and the risk of constipation in pregnant women.

## References

[1] Wuhan J. Gastrointestinal Motility Group of Digestive Disease Branch and Colorectal Group of Surgery Branch of Chinese Medical Association. Chinese Guideline for Chronic Constipation. Chin J Dig. 2013;33(5): 291–297.

[2] Bharucha AE, Dorn SD, Lembo A, Pressman A. American Gastroenterological Association medical position statement on constipation. Gastroenterology. 2013 1;144(1): 211–7. 10.1053/j.gastro.2012.10.029 23261064

[3] Siproudhis L, Pigot F, Godeberge P, Damon H, Soudan D, Bigard MA. Defecation disorders: a French population survey. Dis Colon Rectum. 2006 2;49(2): 219–27. 16362804 10.1007/s10350-005-0249-8

[4] Peppas G, Alexiou VG, Mourtzoukou E, Falagas ME. Epidemiology of constipation in Europe and Oceania: a systematic review. BMC Gastroenterol. 2008 2 12;8: 5 10.1186/1471-230X-8-5 18269746 PMC2258300

[5] Higgins PD, Johanson JF. Epidemiology of constipation in North America: a systematic review. Am J Gastroenterol. 2004 4;99(4): 750–9. 15089911 10.1111/j.1572-0241.2004.04114.x

[6] Jun DW, Park HY, Lee OY, Lee HL, Yoon BC, Choi HS, et al A population-based study on bowel habits in a Korean community: prevalence of functional constipation and self-reported constipation. Dig Dis Sci. 2006 8;51(8): 1471–7. 16832618 10.1007/s10620-006-9087-3

[7] Zhao YF, Ma XQ, Wang R, Yan XY, Li ZS, Zou DW, et al Epidemiology of functional constipation and comparison with constipation-predominant irritable bowel syndrome: the Systematic Investigation of Gastrointestinal Diseases in China (SILC). Aliment PharmacolTher. 2011 10;34(8): 1020–9. 10.1111/j.1365-2036.2011.04809.x 21848795

[8] Dennison C, Prasad M, Lloyd A, Bhattacharyya SK, Dhawan R, Coyne K. The health-related quality of life and economic burden of constipation. Pharmacoeconomics. 2005;23(5): 461–76. 15896098 10.2165/00019053-200523050-00006

[9] Liu W, Liu XH, Fang XC, Zhou LK, Yang XL, Ke MY, et al A Multicenter Epidemiological Investigation On Outpatients with Chronic Constipation in Beijing Area.Chin J Gastroenterol, 2010, 15(2): 95–98.

[10] You JS, Park JY, Chang KJ. A case-control study on the dietary taurine intake, nutrient status and life stress of functional constipation patients in Korean male college students. J Biomed Sci. 2010 8 24; 17 Suppl 1: S41 10.1186/1423-0127-17-S1-S41 20804618 PMC2994376

[11] Devanarayana NM, Rajindrajith S. Association between constipation and stressful life events in a cohort of Sri Lankan children and adolescents. J Trop Pediatr. 2010 6;56(3): 144–8. 10.1093/tropej/fmp077 19696192

[12] Kil SY, Oh WO, Koo BJ, Suk MH. Relationship between depression and health-related quality of life in older Korean patients with chronic obstructive pulmonary disease. J ClinNurs. 2010 5;19(9–10): 1307–14. 10.1111/j.1365-2702.2009.03053.x 20500340

[13] Cheng C, Chan AO, Hui WM, Lam SK. Coping strategies, illness perception, anxiety and depression of patients with idiopathic constipation: a population-based study. Aliment PharmacolTher. 2003 8 1;18(3): 319–26. 10.1046/j.1365-2036.2003.01663.x 12895216

[14] Jung HK, Kim DY, Moon IH. Effects of gender and menstrual cycle on colonic transit time in healthy subjects. Korean J Intern Med. 2003 9;18(3): 181–6. 14619388 10.3904/kjim.2003.18.3.181 PMC4531623

[15] Sandler RS, Jordan MC, Shelton BJ. Demographic and dietary determinants of constipation in the US population. Am J Public Health. 1990 2;80(2): 185–9. 2297063 10.2105/ajph.80.2.185 PMC1404600

[16] Longstreth GF, Thompson WG, Chey WD, Houghton LA, Mearin F, Spiller RC. Functional Bowel Disorders. Gastroenterology. 2006;130: 1480–1491 16678561 10.1053/j.gastro.2005.11.061

[17] Wu JY, Liu XH, Liu W, Ke MY, Fang XY. Investigation of female chronic constipation: a multi-center cross-sectional clinical study. Natl Med J China, 2009, 89(18): 1255–1258. 19595179

[18] Fishman L, Lenders C, Fortunato C, Noonan C, Nurko S. Increased prevalence of constipation and fecal soiling in a population of obese children. J Pediatr. 2004 8;145(2): 253–4. 15289779 10.1016/j.jpeds.2004.04.022

[19] Li ZJ, Yu PL, Shi QK, Jiang ZY, Chu DF, Lv XZ. A cross-sectional study of constipation in elderly people in some urban-rural areas in Beijing. Chin J Geront,2000, 20(1): 1–2.

[20] Xin HW, Zhu LM, Fang XC, Fei GJ, Yao F, Wang LY, et al A Survey on Emotion and Sleep Status in Patients with Functional Constipation. Chin J Gastroenterol, 2012, 17(2): 87–90.

[21] Nakaji S, Tokunaga S, Sakamoto J, Todate M, Shimoyama T, Umeda T, et al Relationship between lifestyle factors and defecation in a Japanese population. Eur J Nutr. 2002 12;41(6): 244–8. 12474067 10.1007/s00394-002-0380-4

[22] Liu ZY, Yang GG, Shen Z, He WY, He F, Yuan YM. Epidemiological survey of constipation in urban people in Hangzhou.Chin J Dig 2004, 24(7): 435–436.

[23] Kan ZC, Yao HC, Long ZP, Liu ZW, Han YS, Zhang ZG, et al The prevalence of adult's chronic constipation in Tianjin and its associated pathogenetic factors. Chin J Dig, 2004, (10): 612–614.

[24] Wei XQ, Chen MH. The epidemiology of functional constipation of Guangzhou residents. Chin J Gastroenterol Hepatol, 2001, 10(2): 150–1.

[25] Guo XF, Ke MY, Pan GZ, Han SM, Fang XC, Lu SC, et al Epidemiological survey and related risk factors analysis of chronic constipation in adults in Beijing, using proportional population sampling, stratified clustering sampling and random sampling methods.Chin J Dig,2002, 22(10): 637–8.

[26] Pare P, Ferrazzi S, Thompson WG, Irvine EJ, Rance L. An epidemiological survey of constipation in canada: definitions, rates, demographics, and predictors of health care seeking. Am J Gastroenterol. 2001 11;96(11): 3130–7. 11721760 10.1111/j.1572-0241.2001.05259.x

[27] Chen LY, Ho KY, Phua KH. Normal bowel habits and prevalence of functional bowel disorders in Singaporean adults—findings from a community based study in Bishan. Community Medicine GI Study Group. Singapore Med J. 2000 6;41(6): 255–8. 11109339

[28] Barbezat G, Poulton R, Milne B, Howell S, Fawcett JP, Talley N. Prevalence and correlates of irritable bowel symptoms in a New Zealand birth cohort. N Z Med J. 2002 10 25;115(1164): U220 12552296

[29] Stewart WF, Liberman JN, Sandler RS, Woods MS, Stemhagen A, Chee E, et al Epidemiology of constipation (EPOC) study in the United States: relation of clinical subtypes to sociodemographicfeatures. Am J Gastroenterol. 1999 12;94(12): 3530–40. 10606315 10.1111/j.1572-0241.1999.01642.x

[30] Tuteja AK, Talley NJ, Joos SK, Woehl JV, Hickam DH. Is constipation associated with decreased physical activity in normally active subjects? Am J Gastroenterol. 2005 1;100(1): 124–9. 15654791 10.1111/j.1572-0241.2005.40516.x

[31] Drossman DA. The functional gastrointestinal disorders and the Rome III process.Gastroenterology,2006,130: 1377–901 16678553 10.1053/j.gastro.2006.03.008

[32] Garrigues V1, Gálvez C, Ortiz V, Ponce M, Nos P, Ponce J. Prevalence of constipation: agreement among several criteria and evaluation of the diagnostic accuracy of qualifying symptoms and self-reported definition in a population-based survey in Spain. Am J Epidemiol. 2004 3 1;159(5): 520–526. 14977649 10.1093/aje/kwh072

[33] Bradley CS, Kennedy CM, Turcea AM, Rao SS, Nygaard IE. Constipation in pregnancy: prevalence, symptoms, and risk factors. Obstet Gynecol. 2007 12;110(6): 1351–7. 18055731 10.1097/01.AOG.0000295723.94624.b1

[34] Fu CW, Xu B, Chen WQ, Luan RS, Zhan SY.A cross-sectional study on the prevalence of depressive, anxiety disorder in outpatients with irritable bowel syndrome and functional dyspepsia in urban China. Chin J Dig. 2006,26(3): 151–154.

[35] Jin JJ, Pan LL. Efficacy of wheat cellulose treatment on 40 cases of women with functional constipation. China Prac Med, 2009, 4(35): 106–108.

[36] Drossman DA, Dumitrascu DL. Rome III: New standard for functional gastrointestinal disorders. J Gastrointestin Liver Dis. 2006 9;15(3): 237–41. 17013448

